# Mandibular Follicular Lymphoma: A Rare Extranodal Presentation With Initial Facial Bone Involvement

**DOI:** 10.7759/cureus.94289

**Published:** 2025-10-10

**Authors:** Catarina Vital, Paula Maria Leite, Mariluz Martins, Pedro Gomes

**Affiliations:** 1 Department of Oral Surgery, Unidade Local de Saúde de São José, Lisbon, PRT; 2 Department of Oral Surgery, Instituto Português de Oncologia do Porto Francisco Gentil, Porto, PRT; 3 Department of Head and Neck Surgery, Instituto Português de Oncologia de Lisboa Francisco Gentil, Lisbon, PRT

**Keywords:** flow cytometry diagnosis, follicular lymphoma (fl), lymphoproliferative disorder (lpd), mass mandibular, oral manifestation

## Abstract

Follicular lymphoma is a common subtype of B-cell non-Hodgkin lymphoma, typically presenting with nodal involvement and, less frequently, in extranodal locations. Primary involvement of the mandible is extremely rare and is often mistaken for odontogenic or inflammatory pathologies, leading to delayed diagnosis. We present the case of a 73-year-old female patient with progressive swelling of the left hemimandible, associated with hypoesthesia of the ipsilateral lower lip. Computed tomography revealed an infiltrative soft tissue lesion with cortical bone erosion and involvement of the inferior alveolar canal. Flow cytometry identified a clonal population of CD20+, CD10+, and BCL2+ B cells, consistent with a diagnosis of follicular lymphoma. Staging with positron emission tomography demonstrated additional extranodal involvement, establishing the disease as Ann Arbor stage IV. The patient was proposed for systemic chemotherapy with rituximab, cyclophosphamide, vincristine, and prednisone, followed by maintenance with rituximab, showing marked clinical and functional improvement after the second treatment cycle. This case highlights the importance of considering lymphoproliferative disorders in the differential diagnosis of persistent mandibular masses. It also underscores the value of flow cytometry in evaluating complex oral presentations and emphasizes the role of a multidisciplinary approach for early diagnosis and effective treatment.

## Introduction

Follicular lymphoma (FL) is a slow-growing (indolent) B-cell non-Hodgkin lymphoma that typically involves lymph nodes and presents with painless lymphadenopathy [1,2]. While nodal involvement is the hallmark of FL, extranodal presentations are uncommon. Primary involvement of the mandible is exceptionally rare and often mimics common odontogenic or inflammatory conditions, which can lead to misdiagnosis and delayed treatment [2-5].

FL accounts for approximately 20-30% of adult non-Hodgkin lymphomas and is characterized by an indolent clinical course with predominantly nodal disease [1]. It typically affects adults, with a median age at diagnosis around 60 years, and shows a slight female predominance. The etiology remains largely unknown, but genetic alterations such as the t(14;18)(q32;q21) translocation, which leads to overexpression of the anti-apoptotic BCL2 protein, play a central role in pathogenesis [1]. Environmental and immunological factors may also contribute, although definitive risk factors are not well established.

FL staging is done according to the Ann Arbor staging system, which is divided into four different stages. Stage I comprises cases with localized disease only. Stage II includes involvement of two or more lymph node regions on the same side of the diaphragm, or localized involvement of a single extralymphatic organ and its regional lymph nodes. Stage III involves lymph node regions on both sides of the diaphragm, which may also include the spleen, or localized involvement of an extralymphatic site. Finally, stage IV corresponds to cases with disseminated involvement of one or more extralymphatic organs, such as bone marrow or viscera [6,7]. Most patients present with advanced-stage disease at diagnosis [8].

Treatment is individualized based on stage, symptoms, and tumor burden. Asymptomatic patients with low tumor burden may be managed with a “watch and wait” strategy. Symptomatic or advanced-stage disease is typically treated with immunochemotherapy, most commonly rituximab combined with chemotherapy agents, such as R-CHOP (rituximab, cyclophosphamide, doxorubicin, vincristine, and prednisone) or R-CVP (rituximab, cyclophosphamide, vincristine, and prednisone), followed by maintenance rituximab in selected cases [8]. Prognosis is generally favorable, with median overall survival exceeding 10 years, although histological transformation to more aggressive lymphomas can negatively impact outcomes [8].

The clinical presentation in extranodal sites like the mandible is often nonspecific, including painless masses, paresthesia, and subtle or absent radiographic changes. These manifestations may mimic odontogenic infections, chronic osteomyelitis, or benign tumors, which contribute to diagnostic delays, impacting staging and prognosis [2,4,5]. Definitive diagnosis requires histopathological and immunophenotypic confirmation, with flow cytometry serving as a rapid and sensitive tool to detect B-cell clonality [9].

This report describes a case of FL diagnosed following mandibular presentation in the context of disseminated systemic disease. The aim is to emphasize the importance of including lymphoproliferative disorders in the differential diagnosis of maxillofacial masses and to reinforce the role of flow cytometry and multidisciplinary management in diagnosis and therapeutic planning.

## Case presentation

A 73-year-old female patient, with a history of total gastrectomy and adjuvant chemoradiotherapy for gastric adenocarcinoma (2015), was referred for progressive swelling of the left mandibular region, with three months of evolution. Hypoesthesia of the ipsilateral lower lip appeared approximately one month ago, and dysphagia developed during the last week.

**Figure 1:**
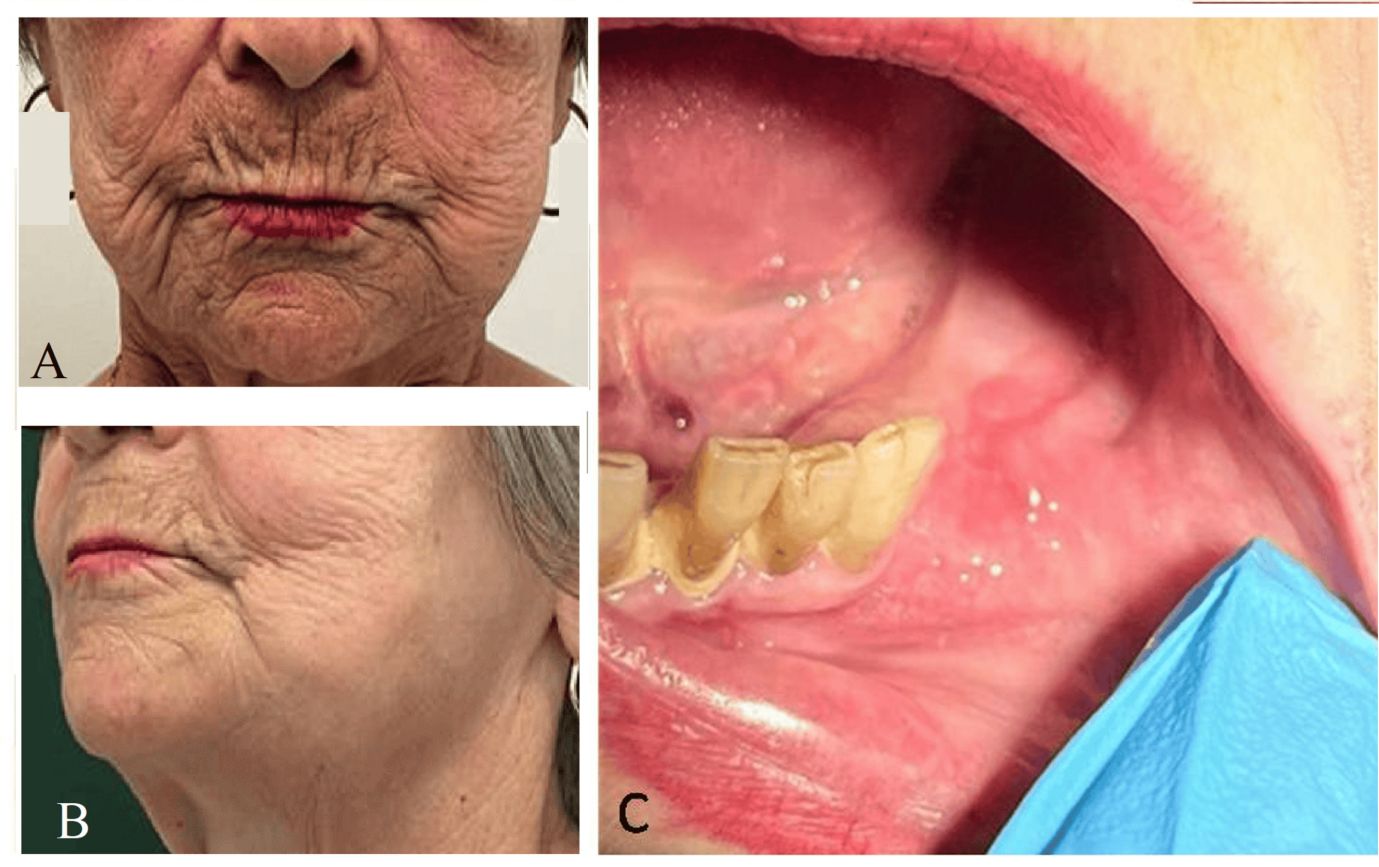
Initial clinical presentation of the patient. (A, B) Frontal and left oblique facial photographs showing asymmetry of the lower third of the face due to swelling of the left mandibular region, without overlying skin changes. (C) Intraoral image of the left posterior mandibular alveolar ridge, revealing a firm, non-ulcerated swelling extending from the retromolar trigone to the region of tooth 33, with no mucosal abnormalities or signs of infection.

On facial examination, there was facial asymmetry with enlargement of the left lower third of the face, without any inflammatory signs, fluctuation, tenderness on palpation, trismus, or dyspnea. Intraoral assessment revealed total edentulism of the upper arch and partial edentulism of the lower arch, rehabilitated with ill-fitting removable acrylic prostheses. There was a firm, painless swelling extending from the left retromolar trigone to the region of tooth 33, with no fluctuation, mucosal changes or palpable cervical lymphadenopathy (Figure 1).

**Figure 2:**
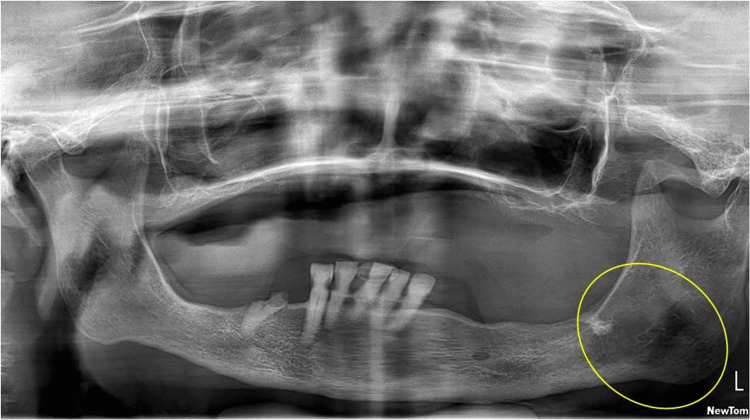
Panoramic radiograph (orthopantomography) showing radiolucent and radiopaque changes in the left mandibular body and angle. A well-defined radiopaque structure, consistent with a retained or impacted root undergoing osseointegration, is observed in the region of the left posterior mandible (highlighted in the yellow circle). Adjacent to this, an ill-defined radiolucent area extends through the posterior mandibular body and angle, associated with cortical thinning and partial involvement of the inferior alveolar canal, suggesting the presence of an infiltrative lesion.

**Figure 3:**
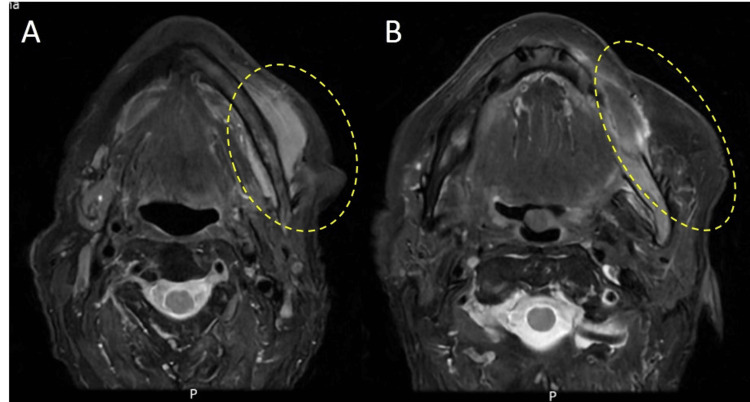
Axial and coronal T2-weighted MRI demonstrating an infiltrative lesion of the left hemimandible. (A, B) Axial T2-weighted images showing a hypointense, diffuse infiltrative lesion involving the left hemimandible (yellow dashed circles), with soft tissue extension and preservation of the cortical outline.

**Figure 4:**
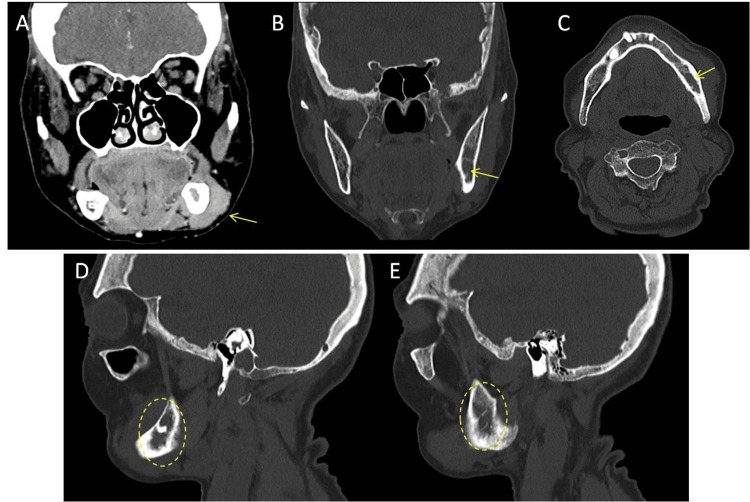
Maxillofacial CT showing an infiltrative lesion of the left mandible, consistent with extranodal follicular lymphoma. The lesion measures 40 × 30 × 30 mm (transverse × craniocaudal × anteroposterior) (A) Coronal soft tissue window demonstrating an expansive, infiltrative mass in the left buccal space (yellow arrow), extending into the mental foramen. (B) Coronal bone window showing partial erosion of the cortical bone and involvement of the inferior alveolar canal (yellow arrow). (C) Axial bone window view revealing lateral cortical disruption and inferior alveolar canal involvement (yellow arrow). (D, E) Sagittal bone window views depicting cortical thickening (suggestive of hyperostosis) and diffuse changes in the trabecular bone of the left hemimandible (dashed yellow circles), with no evident osteolytic lesions.

Orthopantomography showed a radiolucent lesion in the posterior region of the third quadrant, with cortical thinning and partial involvement of the inferior alveolar canal (Figure 2). Magnetic resonance imaging (MRI) revealed a diffuse, hypointense infiltrative lesion in the left hemimandible, sparing the condyle and coronoid process, with cortical bone appearing preserved (Figure 3). In contrast, maxillofacial computed tomography (CT) revealed cortical and cancellous bone erosion, confirming an expansive and infiltrative soft tissue mass extending into the submandibular and masticator spaces, involving the mental foramen and inferior alveolar canal (Figure 4).

**Figure 5:**
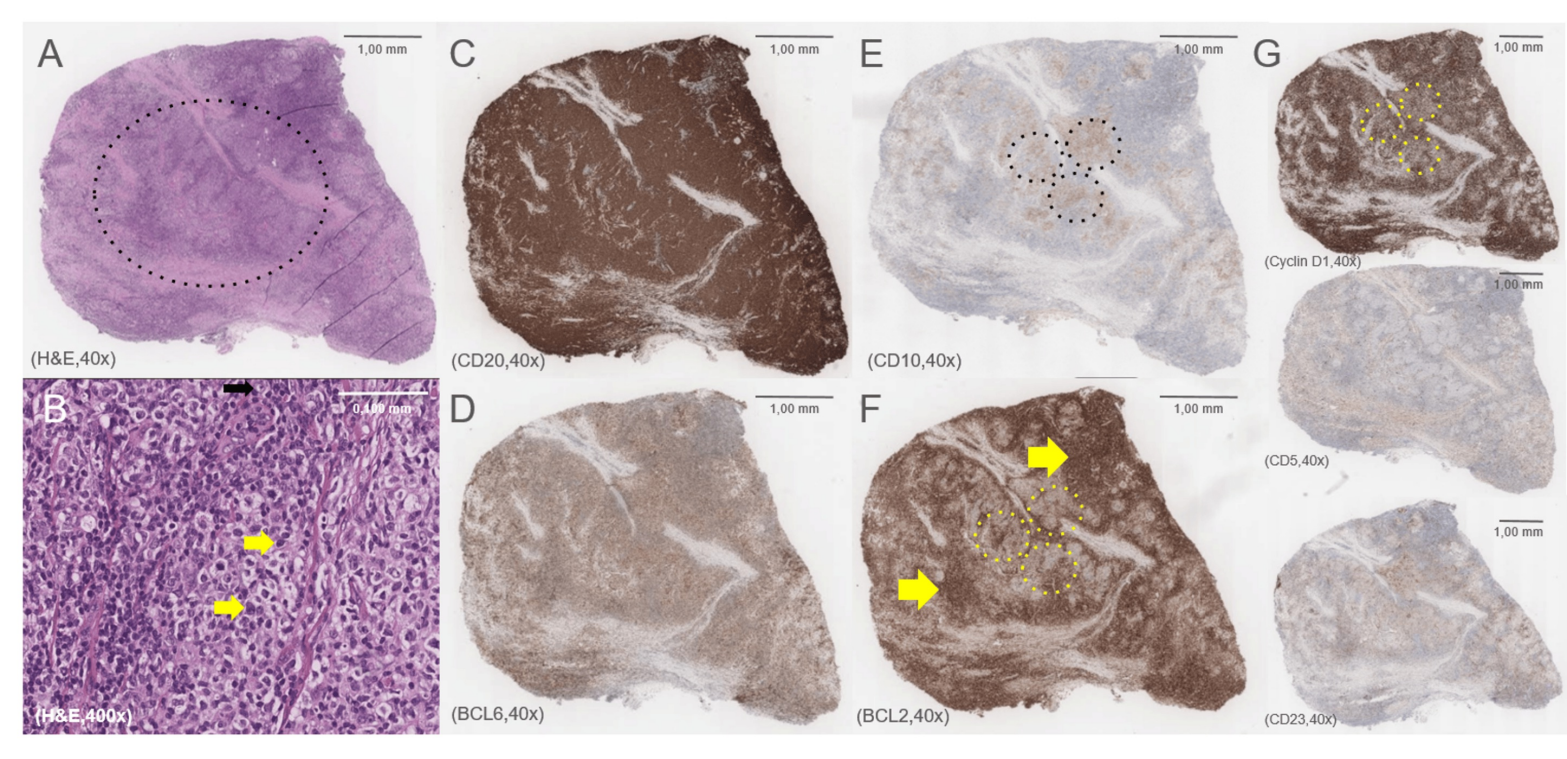
Histopathological and immunohistochemical features of follicular lymphoma in the left mandibular region. (A) Hematoxylin and eosin (H&E) staining (×40) shows effacement of the normal tissue architecture and a vaguely nodular (follicular) growth pattern (black dotted circle). (B) Higher magnification H&E staining (×400) reveals a neoplastic infiltrate composed predominantly of small centrocyte-like lymphocytes with cleaved, irregular nuclei and scant cytoplasm (yellow arrows), interspersed with rare centroblasts (black arrow). The low centroblast count (<15 per high-power field) is consistent with low-grade follicular lymphoma (WHO Grade 1-2), reflecting an indolent clinical course. (C) CD20 immunohistochemistry (×40) shows diffuse membranous positivity, confirming B-cell lineage of the neoplastic population and highlighting the follicular architecture. (D) BCL6 immunohistochemistry (×40) demonstrates heterogeneous nuclear positivity in neoplastic cells, supporting a germinal center phenotype characteristic of follicular lymphoma. (E) CD10 staining (x40) is focally positive in the neoplastic cells (black dotted circles), particularly within follicular structures, supporting a germinal center B-cell origin. (F) BCL2 staining (x40) is negative in neoplastic cells (yellow dotted circles), which can occur in a subset of low-grade follicular lymphomas, but normally highlights the T-cell background (yellow arrows). (G) Immunostaining for Cyclin D1 (yellow dotted circles), CD5, and CD23 (x40) is negative, ruling out differential diagnoses such as mantle cell lymphoma (Cyclin D1+), and chronic lymphocytic leukemia/small lymphocytic lymphoma (CD5+, CD23+), among other B-cell neoplasms. Image Credit: Images and descriptions kindly provided by Dr. Ana Ramos Canastra, Pathology Resident, IPO Lisboa Francisco Gentil

Fine-needle aspiration with flow cytometry showed 18.5% clonal B cells (CD20+, CD10+, BCL2+). Incisional biopsy of the mandibular lesion confirmed morphology and immunophenotype consistent with classic follicular lymphoma (Figure 5). Bone marrow biopsy showed normocellular marrow without lymphomatous infiltration.

**Figure 6:**
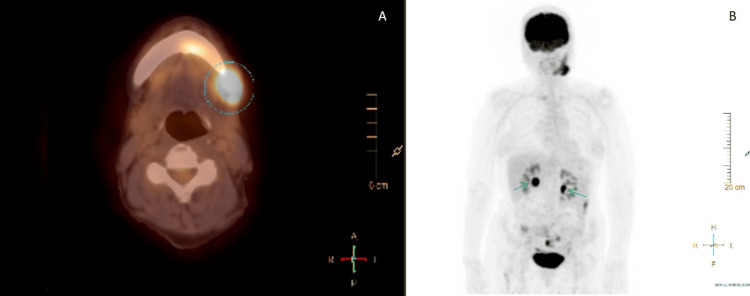
PET-CT scan demonstrating systemic involvement consistent with Ann Arbor stage IV follicular lymphoma. (A) Axial PET-CT image showing increased fluorodeoxyglucose (FDG) uptake in the left buccal space and mandibular body (blue circle), corresponding to the primary mandibular lesion, with a maximum standardized uptake value (SUVmax) of 10.5 — suggestive of metabolically active lymphoproliferative disease. (B) Whole-body maximum intensity projection (MIP) reveals additional FDG-avid sites, including low-grade diffuse uptake in the sternum and right proximal femur. Multiple foci of low-to-moderate FDG uptake are also observed in the gastrointestinal tract, particularly in the transverse colon (green arrows), consistent with multifocal extranodal involvement. These findings supported the diagnosis of disseminated disease and were crucial for staging.

Positron emission tomography (PET-CT) revealed pathological uptake in the left oral space and along the ipsilateral mandible, as well as in additional locations (duodenum, sternum, and femur), establishing Ann Arbor stage IV disease (Figure 6).

The patient was discussed in a multidisciplinary meeting and proposed for chemotherapy with the R-CVP regimen (rituximab, cyclophosphamide, vincristine, and prednisone) followed by rituximab maintenance. Regression of the swelling and neurological improvement were seen after the second cycle, and the patient remains under follow-up in the Hematology clinic.

## Discussion

Mandibular involvement by follicular lymphoma is rare and poses a diagnostic challenge, particularly due to the clinical overlap with odontogenic and inflammatory pathologies [2-4]. The absence of early cortical destruction and the indolent nature of the tumor contribute to diagnostic delays [5].

Hypoesthesia of the mental nerve is a red flag for malignancy and should prompt further investigation [5,10]. Given the patient’s previous history of gastric adenocarcinoma, the possibility of a metastatic mandibular lesion was also considered. Although oral metastases are rare, the mandible is the most frequent site, and gastric adenocarcinoma has been described as a potential primary tumor [11]. Careful clinicopathological correlation and immunohistochemical analysis were essential to exclude this hypothesis in the present case.

In this case, flow cytometry played a key role, enabling a rapid and definitive diagnosis from minimally invasive samples [9]. PET-CT was essential for staging and defining the therapeutic strategy [7,12], and the multidisciplinary approach involving Oral Medicine, Radiology, Pathology, Hematology, and Oncology was crucial for optimizing the patient’s clinical management.

The prognosis of FL depends on stage and therapeutic response and is generally favorable in cases sensitive to combined immunochemotherapy [6]. However, there is a risk of histological transformation into aggressive lymphoma, warranting long-term surveillance [13].

This report contributes to the medical literature by documenting a rare oral manifestation of FL, reinforcing the need to maintain a broad and multidisciplinary differential diagnosis for persistent mandibular masses.

## Conclusions

Although follicular lymphoma is common among B-cell non-Hodgkin lymphomas, it rarely has its initial presentation in the mandible. This case underscores the importance of considering lymphoproliferative disorders in the differential diagnosis of persistent mandibular masses, especially when associated with neurological deficits.

Diagnosis requires the integration of clinical, imaging, and immunophenotypic data, with flow cytometry serving as a valuable tool. Timely recognition, combined with a coordinated multidisciplinary approach, is essential for improving patient prognosis and advancing our understanding of the rare extranodal manifestations of follicular lymphoma.
